# 160. Measuring the Impact of PCR-based MRSA Nares Screening on Vancomycin Use and Duration of Vancomycin Among Pneumonia Patients at an Academic Medical Center

**DOI:** 10.1093/ofid/ofad500.233

**Published:** 2023-11-27

**Authors:** Joseph Stromberg, Travis Jones, Nikolaos Mavrogiorgos, Ashley H Marx

**Affiliations:** University of North Carolina, Durham, North Carolina; UNC Medical Center, Durham, North Carolina; University of North Carolina Medical Center, Chapel Hill, North Carolina; University of North Carolina Medical Center, Chapel Hill, North Carolina

## Abstract

**Background:**

The benefits of PCR-based MRSA nares screening for de-escalation of pneumonia therapy are well-established. In late 2022, UNCMC introduced a PCR-based MRSA nares screen for inpatient use, replacing a chromogenic culture-based screen. The improved turnaround time of the PCR-based test was hypothesized to increase adoption of screening for empirical de-escalation. This project assessed adoption of this diagnostic and measured impact on patients with pneumonia who comprise a cohort followed by the ASP using a visualization dashboard.

**Methods:**

A report was generated of patients with admission ICD-10 codes compatible with pneumonia for January-March of 2022 (pre-PCR) and 2023 (PCR). Electronic medical records of included encounters were reviewed to collect respiratory culture data, validate vancomycin exposure days within the context of the patient’s renal function, and confirm presence of clinical pneumonia. For 2022 data, MRSA screen results were manually collected from the medical record, while for 2023 data, encounters were matched with MRSA PCR results. Fisher’s exact was used to analyze categorical data.

**Results:**

46 and 68 patients, respectively, were included for 2022 and 2023. There was a trend increase in the percentage of patients who were screened for MRSA (28% to 51%, p = 0.68; Table 1). Median turnaround time decreased from 2,022 min to 112 min. The frequency of respiratory sputum cultures, MRSA positivity, and empiric vancomycin therapy were similar. Median duration of vancomycin exposure decreased among all vancomycin recipients (3 to 2 days; Table 2). The percentage of vancomycin recipients who received just one day of coverage increased from 22% to 46%, a trend observed regardless of MRSA screening (Figure 1).

**Figure:**
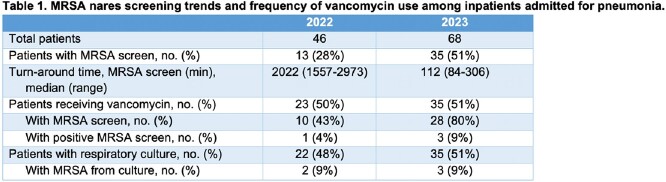

**Figure:**
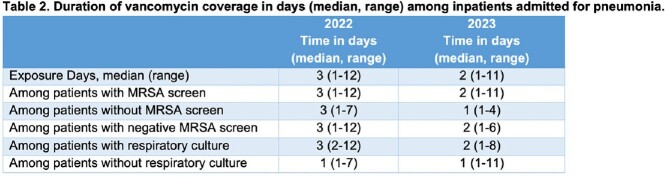

**Figure:**
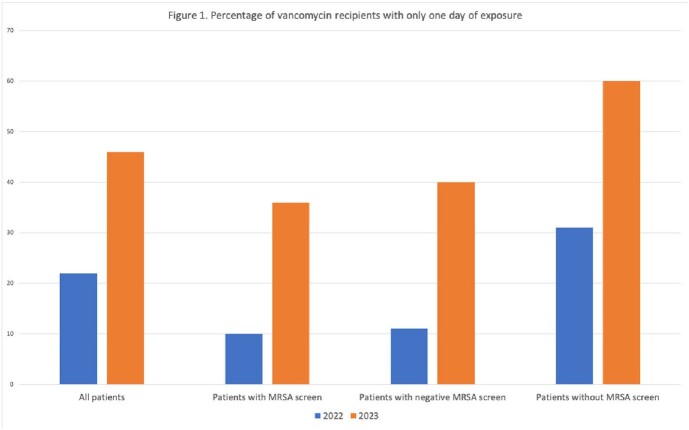

**Conclusion:**

The PCR-based MRSA nares screen for inpatients with pneumonia has trended toward wider adoption compared to the prior screening method, and PCR has been associated with decreased durations of vancomycin therapy. However, similar trends were present in patients who did and did not undergo MRSA screening, suggesting both the new MRSA screen and broader institutional factors have contributed to a shift away from empirical anti-MRSA coverage for pneumonia.

**Disclosures:**

**All Authors**: No reported disclosures

